# Blueprint for destigmatizing depression and increasing openness to treatment among adolescents using brief social contact-based videos: a qualitative study

**DOI:** 10.3389/frcha.2024.1386284

**Published:** 2024-04-11

**Authors:** Madeline DiGiovanni, Samantha E. Jankowski, Lisa B. Dixon, Andrés Martin, Doron Amsalem

**Affiliations:** ^1^ Child Study Center, Yale School of Medicine, New Haven, CT, United States; ^2^ New York State Psychiatric Institute and Department of Psychiatry, Columbia University Vagelos College of Physicians & Surgeons, New York, NY, United States; ^3^ Simulated Participant Program, Teaching and Learning Center, Yale School of Medicine, New Haven, CT, United States

**Keywords:** depression, stigma, adolescents, brief video intervention, qualitative methods

## Abstract

**Background:**

Depression is a major public health concern for adolescents, who exhibit low rates of connection to care despite significant needs. Although barriers to help-seeking such as stigma are well documented, interventions to address stigma and to increase help-seeking behavior are insufficient. Dissemination of short videos in social media offer a promising approach, but designing effective stimuli requires better insight into adolescents’ perspectives of their own experiences, barriers, and possible interventions.

**Methods:**

We conducted 20 semi-structured interviews with adolescents recruited online via RecruitMe, a Columbia University clinical research registry, to explore their perceptions of depression stigma, barriers to care, the role of schools, and the role of brief video interventions. Thematic analysis guided our analytic approach.

**Results:**

We developed a model consisting of three major domains: (1) Barriers to Help-Seeking, which depicts participants *debating their locus of control*, naming s*ocial, parental, and peer stigma*, and *acknowledging systemic barriers*; (2) Importance of School Systems, in which participants elaborated on the role of schools *filling unmet needs* and the importance of *being taken care of*; and (3) Role of Social Media Videos, characterized by adolescents who are *seeking legitimacy, finding cultural authenticity,* and working towards *creating an accessible blueprint* for better mental healthcare.

**Discussion:**

We provide insights into adolescents’ perceptions of help-seeking for depression and what nuances they would hope to see reflected in future interventions, most notably school-based interventions and peer social media videos. Our study offers a steppingstone towards the creation of brief, social contact-based video interventions focused on destigmatizing depression and promoting openness to treatment among adolescents.

## Introduction

Depression is a major public health concern for adolescents, with prevalence nearly doubling in the past decade. Approximately 1 in 6 adolescents currently experience a major depressive episode in a 12-month span (1). Adolescent depression is associated with multiple adverse outcomes, including failure to complete school and future unemployment (2). Longer duration of untreated depression is associated with increased depression severity at follow-up, worse cognitive performance, and poorer prognosis (as measured by degree of change in depression scores after two years) (3, 4).

Despite these sobering facts, few adolescents seek help for depressive symptoms—even with sufficient baseline mental health literacy—in part due to stigma (5). Mental health stigma is defined as negative beliefs, stereotypes, and fears of individuals with mental illness (6), which can be internalized to create self-stigma (7). Studies demonstrate that help-seeking for depression is impaired by perceived stigma, with fear or expectation of discrimination against people diagnosed with mental illness, leading to treatment delay or avoidance (8, 9).

Stigma has been theorized to be particularly impactful for adolescents given the developmental considerations of identity formation, self-image, and autonomy. However, the impact varies depending on support seeker or provider type (5, 10). Adolescents are acutely aware of peers’ perceptions and report depression stigma from peers more frequently than from adults, describing perceived negative attitudes about depression labels or symptoms as well as social isolation and even harassment from peers (11). Perceptions of peer depression stigma also engender perceived stigma of seeking help: Being “too embarrassed by what other kids would say” significantly reduces adolescents’ willingness to utilize mental health services (12). Because of the unique susceptibilities of this population, targeting self- and peer stigma against mental illness can help reduce treatment avoidance and increase access to much-needed mental healthcare (13).

To increase help-seeking for depression in adolescents, brief video interventions for social contact offer a promising frontier. Jankowski et al (14) summarize the impactful elements; effective videos portray hopeful stories that counteract negative stereotypes, are conveniently sharable and a manageable length, and equally as effective as in-person social contact. For adolescents specifically, prior investigations demonstrate impactful reductions in stigma and increases in help-seeking intent with brief video interventions for depression (15), especially when video scripts account for specific aspects of the protagonist's identity such as race (16). These studies, among others, support emphasizing the intersections of identity and mental illness to augment effects for stigma reduction and help-seeking intent (15–17).

Studies exploring how to thoughtfully craft authentic videos are limited (18), especially for adolescents. Further, despite the significant need for interventions to increase help-seeking in this vulnerable population, most existing interventions are adapted from studies of adults rather than incorporating adolescent perspectives. This study aims to fill that gap by providing detailed insights into adolescents’ experiences to inform the authenticity of future videos. First, we aim to capture the intersections of adolescent identity and mental illness through qualitative investigation of youths’ perceptions, lived experiences, and attitudes toward the strengths and limitations of existing video interventions. Prior work suggests that, in addition to perceived stigma, barriers to help-seeking include adolescent-specific factors related to their status as dependents (e.g., the importance of parental or family beliefs, desire for increased independence) (19). Accordingly, we target concepts relevant to adolescence, such as impact of parental beliefs and perceptions of teen-specific barriers to help-seeking. Second, we aim to evaluate adolescents’ perceptions of school-based supports and social media platforms as the two main environments for teenagers where these videos could be disseminated. In addition to the work supporting video interventions above, prior work demonstrates that many interventions for depression stigma and help-seeking are classroom-based (19). Accordingly, we target not only perceptions of the efficacy of video-based interventions, but also perceptions of school's role in depression destigmatization and mental health supports.

## Methods

### Participants and procedure

We conducted individual interviews from March to June of 2023 via HIPAA-compliant Zoom. Participants were recruited via RecruitMe, an online Columbia University clinical research registry, and were identified via voluntary and purposive sampling. Participants could find the registry via Internet search, word of mouth, local brochures, or direct physician referral. The study was advertised as an opportunity to share thoughts on depression and mental health-related discrimination, advertised under the topics of “depression” and “pediatrics.” Potential participants were informed that the research aims were to explore discrimination surrounding depression and barriers to care, with the ultimate goal of using the findings to create videos that reduce such discrimination. The advertisement clearly outlined what participation would entail, including engaging in individual interviews lasting approximately 45 min, conducted remotely via HIPAA-compliant Zoom. It emphasized the compensation of $25 for participation, assured confidentiality of personal identity while noting the collection of limited demographic information, and specified eligibility criteria, including age, language proficiency. The need for parental permission was highlighted. Research staff obtained written consents from study participants and their parents, following an initial screening phone call to verify eligibility for the study (i.e., ages 14–18, English speaking). We provide a list of guiding questions in Appendix 1; all questions were non-mandatory, though no participant declined to answer any question. Participants were asked about perceptions of depression, depression stigma, barriers to care, and school resources before viewing a one-minute, Tiktok-style first-person perspective video of a young teenage girl sharing her experience with depression and seeking help. In the video, the girl describes her symptoms of anhedonia (particularly for her favorite activities) and social withdrawal, and discloses that the onset of suicidal ideation was what motivated her to seek help and speak to her parents and pediatrician. She then briefly outlines the process of being diagnosed and starting medication, before closing with a brief summary of symptomatic and functional improvement and encouragement for others who are struggling to seek help as well. Participants then offered feedback and suggestions for future videos. Interviews were conducted collaboratively between three authors (DA, SJ, MD), with DA as lead interviewer. The project was approved by the Institutional Review Board of the New York State Psychiatric Institute. Participants were compensated $25 for study participation.

### Qualitative analysis

SJ (a female and clinical psychology doctoral candidate) and MD (a female and medical student) transcribed digital audio files utilizing AWS transcription service, an artificial intelligence tool from Amazon, prior to analysis using NVivo (2020 Release) software and Microsoft Word. We utilized thematic analysis to identify overarching meaningful aspects of participants’ experiences (20, 21). Three authors (DA, SJ, MD) independently identified salient themes from the transcripts before consolidating shared codes towards a final framework, triangulated and verified by all coders. We selected an inductive approach to data analysis given the importance of capturing adolescents’ experiences authentically, reflecting the goal of this study, rather than relying on second-hand report from prior literature. Iterative meeting memos ensured theoretical sufficiency prior to the final interview (22). Participants did not preview transcripts or the final framework during analysis. Multiple quotes supported each code, and study design and manuscript preparation followed best practice guidelines for qualitative research (23, 24). Given the authors’ varying experiences in mental health research, the team considered prior assumptions throughout the study as part of their reflexive stance.

### Reflexivity

All authors have varying experience in mental health training, qualitative research, and education. Reflexivity is particularly important for the three study coders: MD is a biracial female medical student with several years’ experience in qualitative methods, a career interest in child and adolescent psychiatry, and prior employment as a high school special education teacher; the latter detail was openly shared with participants when relevant. This author developed Part 3 of the interview guide (see Appendix 1) with care to avoid leading questions. DA is a white male child and adolescent psychiatrist and stigma researcher with several years’ experience in qualitative and quantitative methods, and prior employment as an evening school teacher. SJ is a white female clinical psychology doctoral candidate with several years of experience in conducting psychological treatment and research in youth with early psychosis and stigma. Triangulation and open conversation between all three interviewers, as well as with the two non-interviewing study authors, helped to minimize bias or preconceived assumptions based prior experiences.

## Results

Twenty adolescents completed individual semi-structured interviews, lasting approximately 20–40 min each. Attrition included only one participant after recruitment and consent who did not appear for the scheduled interview and was unable to be reached subsequently. Mean age was 16.5 years old (range 15–18). Our sample included 12 girls, seven boys, and one non-binary participant. Nine participants self-identified as Black, eight as white, and three as multiracial. In transcript analysis, participants were evenly split in statements identifying as having current or past depression. Of the 10 participants not self-reporting a label of depression, gender split was also equal. Within participants that did self-report a label of depression, seven were girls, two were boys, and one was non-binary. There were no repeat interviews.

Our analysis identified three domains, with two to three themes each (Tables 1–3). Each theme was supported by quotes from more than one participant. In (1) Barriers to Help-seeking, we characterize participants’ reflections on perceptions of depression and stigma: Adolescents grappled with questions of identity and moved beyond social stigma when considering barriers to care, offering broader reflections to support help-seeking. In (2) The Importance of School, we address the question of school's role in destigmatization and support: Adolescents identified school as a valuable but limited setting for mental health resources and education. Finally, in (3) Role of Social Media Videos, we identify participants’ perceptions of and desired next steps for video inteventions: Adolescents described videos as a powerful form of identification and encouragement that should be detailed and customized to the target demographic. Domains 1 and 2 support Domain 3 by articulating insight into adolescents’ perceptions of what factors or resources influence their likelihood to seek help for depression, providing concepts that can influence the effectiveness of future social media video interventions.

**Table 1:** Domain I: barriers to help-seeking.

Theme	Representative quote
Debating the locus of control	“The hardest thing for me to face is I didn't want to be labeled as depressed because to me that almost felt more permanent. Like I didn't feel like if someone was to label me as depressed, they meant I was stuck with it.” [*P4*]
Facing social stigma	“They feel that these young people have character flaws. They're not strong enough. It's a sign of weakness for them in dealing with stuff.” [*P19*] “Since it isn't a visible disability, like being in a wheelchair or needing special accommodations for your physical health, it's like, well… how do we know you're telling the truth? Like, are you just being attention-seeking and all of that?” [*P2*]
Acknowledging systemic barriers	“As a teenager, a lot of kids don't really have access to health care because one, just being a minor and two, I feel like at least in public schools—because I go to a public school—there isn't much resources for it to begin with. And I know like a lot of kids, they will often try to go to their guidance counselor for help and then their guidance counselor will just call their parents, even though they don't want their parents involved in the situation.” [*P6*]

**Table 2:** Domain II: the importance of school systems.

Theme	Representative quote
Filling critical needs	“Another thing is that every school should include or maybe have a class or maybe have a liaison whereby everybody can be taught about… how to take care of themselves when it comes to mental health issues. So that class can be just be made even once in a week or twice in a week for everybody to attend so that we can be given teachings or maybe some lessons…” [*P15*]
Being taken care of	“They should definitely provide resources because some people don't have resources at home or they're afraid to ask for help from, like, their parents or family. And for some people, I think school… serves as a safe space… that there's resources and people you can talk to at school is helpful.” [*P7*]

**Table 3:** Domain III: the role of social media videos.

Theme	Representative quote
Seeking legitimacy	“Even when I feel like really sad or I feel really bad, I'll ask myself, like, I—like, I'm not supposed to be feeling like this because I don't have real problems. Like, there's people who have real problems, there's people who are homeless, there's people who they don't have like food.” [*P9*] “I feel like they do this because they don't want to be associated as…. being weak, you know? They don't want to—or rather they feel like no one is going to really pay attention to them or people may think that they are really maybe overreacting, exaggerating, probably making up scenarios and stuff that aren't really there.” [*P17*]
Finding cultural authenticity in videos	“It was great to see that it was a girl just like me. I feel like I would relate more to a girl because our problems are almost similar. I also feel like maybe as a Black person… our experiences may be a little bit different. So, maybe I would relate more to a person from my race. But also, I feel like just having like someone like a woman, it makes me feel safer, because we might go through like the same challenges, especially in high school.” [*P9*]
Creating an accessible blueprint	“When you come to see someone's video of depression and how they came about it and how—if they managed it, it gives you that kind of… I may say hope or motivation to work on yourself also and overcome what you're facing. And also, it can give you an idea, like you can use the method he used or they used, if you are maybe clueless or if your idea isn't working, you can gain some idea from the videos, and you may find it working for you.” [*P14*]

### Domain I. Barriers to help-seeking

Responding to questions about stigma and barriers, adolescents volunteered commentary that reflects multiple levels of perceived stigma: self, interpersonal, and structural. Within themselves, adolescents grappled with the permanence of a stigmatized identity, wondering to what extent the label of “depression” defined them, thereby influencing their motivation to seek help. In addition to internalized identity, stigma from others played a large role in participants’ reflections on help-seeking, reflecting a more interpersonal lens on stigma. Adolescents then pointed beyond social stigma towards larger systemic barriers, such as lack of education or inadequate therapeutic resources, reflecting structural views of stigma.

#### Debating the locus of control

Participants characterized multiple perspectives of depression, wondering in particular about the condition’s permanence and the utility of seeking help, either for symptom resolution or temporizing management. If one's depression is real, what responsibility exists to mitigate it? For some, depression is a core identity, influenced by genetic inheritance or other happenstance, modifiable but not entirely eradicable: “It's a chemical malfunction in your brain and… it's not something you can control or change about yourself. And there's nothing wrong with you—it just means you need help” [*P8*]. For others, depression is a temporary response to individual choices or circumstances that make people “not themselves,” and it can be resolved with enough support and effort: “It's something that's gonna be there, but you're gonna be able to get away from it… You might just become back to your normal self” [*P12*]. Both narratives impacted perceived stigma (in this case, predominantly self-stigma), casting depression as either as an inherent malfunction or a temporary weakness, as well as help-seeking behavior, influencing one's perceived urgency for seeking support. For those who viewed depression as temporary, seeking help was beneficial in resolving the symptoms, but not always necessary: “Depression might die off you” [*P12*], because it's “a passing cloud that can be dealt with” [*P19*]. When considering the impact of peer narratives of depression, participants wanted to feel congruence between their own and their peers’ perspectives of depression.

#### Social, parental, and peer stigma

Reflecting the judgments of others, participants emphasized shame and fear as significant barriers to seeking care, demonstrating the power of interpersonal stigma: “Like, oh, you're just lazy… or you're dark and can't be interacted with, like it's contagious” [*P5*]. The stigma contributed to intense privacy concerns: Confidentiality was paramount for adolescents and an oft-mentioned consideration for seeking help, for fear of judgment from others; adolescents expressed particular concern about having private information shared with their parents by other adults. Participants also cited specific stigmas from parents, including the idea that children should not be depressed because their parents worked to make their lives easier, revealing a burden of responsibility and guilt among participants: “They don't feel like mental issues should be something, because we have a much better life” [*P10*]. Participants also felt judged by their parents for having trivial concerns, despite feeling like “the things that I'm experiencing right now are like the biggest problems that I've experienced in my life so far” [*P9*]. Whether actual or anticipated, parental stigma was a significant element of participants’ responses, and one that spoke uniquely to the experience of adolescence.

From peers, participants noted differential stigma based on public vs. private engagement in conversations about mental illness, with a suspicion that peers “laugh at” mental health due to discomfort with public vulnerability: “It's vulnerable… so instead of choosing to talk about it, people just call it stupid” [*P7*]. Simultaneously, increased public discussion about mental health caught participants in a paradox: Reduced stigma leads to the normalization of depression, but also increased scrutiny of whether one truly fits the label, creating an additional stigma of perceived inauthenticity: “It's a time for everyone to have mental illness and to just like brag about having depression or ADHD, so like the people who are really suffering… may shy away from seeking help because they don't want people to think they're just doing it for attention or joining a trend” [*P9*]. When considering possible peer interventions for stigma reduction, such as through the videos discussed in interviews, participants therefore carefully weighed the pros and cons of sharing such a personal story with a wider audience, with some preferring the use of fictional accounts told by actors rather than true lived experience told by peers. Participants also suggested that negative perceptions of depression originate from a lack of understanding or insufficient mental health literacy; this may reflect the consequences of reluctance to discuss depression publicly as described above.

#### Acknowledging systemic barriers

Participants hypothesized that limited mental health literacy in the general public results in deprioritization of mental health support, creating structural barriers to help-seeking. Because others misunderstand depression, necessary resources are inaccessible or nonexistent: “[People] just think, oh, you're sad. Okay. What am I supposed to do about that? But if people knew that it's like, more than that, like the other symptoms and all that stuff, I think it would be more helpful” [*P1*]. Specifically, participants noted that getting help sometimes necessitates going beyond the usual recommendations to speak up and reach out, naming structural supports as a solution. For example, adolescents desired access to mental healthcare independently of one's parents: “I don't know if you can get like a therapist as, like, being under 18 without your family's consent” [*P7*]. Others named financial support and academic accommodations as resolving stressors contributing to depression. Students willing to use school-based resources noted the barrier of insufficient support staff: “There's also a school counselor. but sometimes I just feel like they're overworked or overwhelmed and sometimes they won't even remember you or like your specific stories because they're dealing with, like, the entire student body” [*P9*]. These barriers reflect participants’ recognition of structural stigma, and participants hoped for future interventions, such as social media videos targeted in this study, to include more concrete resources or recommendations for adolescents to access mental healthcare.

### Domain II. The importance of school systems

Participants emphasized the importance of school systems in providing both mental health education and concrete support resources, noting that schools both have a duty toward and are a good fit for these responsibilities. Reflections on adults’ shared responsibilities highlighted a desire to be taken care of, in tension with the hunger for increased independence.

#### Filling critical needs

Many participants named school as a key site for intervention, alongside the Internet, social media, and hearing from peers first-hand. Students named existing school strategies to bolster student mental health and increase awareness, including displaying informational posters, discussing mental health in health class, and staffing school therapists, social workers, and guidance counselors. Regular contact was positively received: “There's a mindful Monday, a weekly and monthly check in where they ask, how are you doing? Do you need support with school or home? The guidance counselors check in on you pretty frequently” [*P11*]. Simultaneously, many wished that schools could do more to both educate and support, which was viewed as part of school's responsibility: “A school's total responsibility [is] to support them mentally, not educationally alone” [*P13*]. Suggestions included peer support programs, joint awareness programs for kids and parents, mental health syllabi, and mental health-related elective or seminars. Peer interventions were particularly desirable, because they increase a sense of belonging and reduce the age or experience differential between the adolescent and their supporter: “When you talk to your peers, like the way you talk to your best friend, you're just open… like you feel understood” [*P10*]. The beneficial aspects of school environments, such as being able to disseminate better mental health information and connecting participants with an understanding peer community, echoed participants’ commentary on the value of potential social media video interventions, which we analyzed as targeting similar goals. Consequently, when considering potential next steps for de-stigmatizing depression and increase help-seeking behavior, adolescents identified concordant opportunities across in-person and virtual environments (i.e., school and social media, respectively).

#### Being taken care of

Adolescents viewed school as a good place to accomplish these goals because they perceived school as somewhat of a parental surrogate based on hours of supervision: “We spend so many hours in school and then a few with our parents before we go to bed or come back to school. So, I think they have a bigger chance… because we spend more time with them” [*P10*]. Although academics did contribute to some participants’ distress, school itself was not a primary stressor. but rather a safe haven, particularly from students with limited support from home. Part of school's perception as a safe haven lay in the presence of peers, who were perceived to hold more generationally-congruent attitudes towards mental illness compared to adults.

Attitudes towards family and school's shared responsibilities revealed a wish to be taken care of, alongside the independence participants desired in order to access formal mental health resources without their parents: “It's up to other people to help you and you shouldn’t have that responsibility to make yourself better,” noted one participant, who added that “it's been really helpful when [adults] take the reins… and help guide me through it” [*P8*]. Consequently, school figured as a nurturing environment in which to learn, grow, and be cared for. Simultaneously, participants recognized the limitations of schools’ psychosocial role, acknowledging that not every staff member has the sufficient training or time given the academic demands. Participants acknowledged that mental healthcare is a “collective responsibility” shared between parents, school staff, and other community adults: “It's the job of the whole community” [*P14*].

Together, these findings represent tensions in participants’ attitudes towards sources of support. Peers can be acutely judgmental, yet a valued source of validation instead of adults’ misunderstandings of their struggles. School can create additional psychological demands, but also offer belonging and respite from home. Parents can seem unsympathetic, yet simultaneously offer consistency, safety, and access to care. Per some participants, school itself could provide a path to ease these tensions by engaging all parties in psychoeducation:

“Let’s say like a mental health awareness program, like a day that is set aside for parents and students to meet, then they are just educated about mental health and its importance and the way it can be implemented…. They may have appointed some student leaders who can act as peer counselors…. Then if you fear going to the counselor directly, you can trust and approach your fellow classmate or your fellow student to whom maybe you may trust more and may understand what you are going through more.” [*P14*]

### Domain III. Role of social media videos

In their assessments of stigma and opportunities for better care, adolescents revealed a deep uncertainty about depression's implications for core identity—reflecting opportunities for adjustment of future video scripts. Adherence to a “depression identity” (or not) informed a narrative's legitimacy and therefore its relatability, a key factor in adolescents’ interpretation of videos as encouraging for help-seeking behavior, particularly within specific cultural demographics. Participants named videos and social media as effective vehicles for this authentic representation, reinforcing the importance of relatable and accessible peer support.

#### Seeking legitimacy

To inform future video interventions, we identified that perceived validation of one’s depression experience by peer narratives in videos helped ease acceptance of seeking help in the future, making legitimization or validation a key component to consider for future interventions. Reflecting earlier commentary about stigma and identity, adolescents painted a complex picture of belonging, paradoxically casting a stigmatized label as a sometimes-exclusionary in-group with implicit qualifying criteria. Participants struggled to reconcile the idea that depression has prerequisites [“Some people might feel that if they don't have that family background, then the depression doesn't make sense for them” (*P18*)] with an open acceptance of the diversity of illness [“Mental illness can look like anything. It can look like a person like me, or… like my little brother, or like my grandmother…” (*P11*)]. The possibility of “faking it” or “just doing it for attention” was a particularly sensitive accusation, suggesting a deep emphasis on authenticity: “It’s kind of become like a trend. I guess a lot of people—I wouldn't say romanticize… but, like make it seem like something that it's not” [*P3*]. To reduce this discomfort, participants were adamant about the difference between sadness and depression: “People are like, I'm depressed, when they're trying to say they're sad. And I'm like, those are two very different things” [*P4*]. Hearing externally-accepted narratives of depression that resonated with their own helped to “legitimize” participants’ experiences, validating that their struggles also merited seeking help: “This person got help, so… so can I, you know?” [*P2*]. For participants who sense trivialization or dismissal from parents, feeling validated by peer narratives also offered a way to elevate their voices and concers: “I would love my parents to know that depression is not just about money and bills” [*P9*].

#### Finding cultural authenticity in videos

To find these validating stories, adolescents appreciated video narratives from peers. “If they're talking about an experience with mental health that I've experienced similar to, I am going to connect to that,” shared one participant [*P4*]. Participants requested tailoring the video script or protagonist to increase authenticity, especially for participants who self-reported lived experience with depression, who felt the protagonist was sugarcoating her experience. When elaborating on what could increase authenticity, participants emphasized maintaining a realistic tone and avoiding listing symptoms in a stereotypical way: “The tone of the voice sounded a little bit condescending… like a little bit overly peppy… It should sound like it's coming from a teenager” [*P5*]. In fact, perceived authenticity and relatability was the predominant concern in participants’ reflections on social media videos. Participants felt very strongly that future videos should strike the right balance of emotion, with video protagonists being neither overly stoic nor overly caricatured: “It doesn't seem like she's talking from her heart. Like, it feels kind of like… put on, like an act” [*P3*]. Participants also sought relatability in the amount and type of information provided by videos, requesting either more or less detail depending on their satisfaction with the protagonist's portrayal. Above all, participants reported that video narratives should feel realistically detailed rather than generic: “I feel like every video I've seen about depression has been that stereotypical, like, if you went through and googled depression and you read all the symptoms, and then you had a person say that's how they felt” [*P4*]. In order to feel a sense of trust or efficacy in future video stimuli, participants therefore requested careful stimulus design to take affective and informational authenticity into account.

Multiple participants also offered commentary on how their race, gender, or other identities intersected with experiences of stigma and help-seeking. Some felt that gender-specific concerns created unique stressors during adolescence, noting that “I don't think… a boy… would understand what a teenage girl would go through in high school” [*P9*]. Male participants commented on the intersection of stigma and masculinity: “Society believes… that as a man, you should always be strong [*P16*]. Black participants noted that while their personal experiences were not monolithic and they had very supportive families, the stereotypes about Black communities were prominent: “Those notions of—Black people are tough and do anything… They create the barriers of them getting help when they're really—actually they're really in need” [*P19*]. Therefore, when brainstorming new video narratives, participants suggested that protagonists match the target audience’s demographics: “There's other different problems that I may experience as a Black teenager in high school that maybe I would only relate to with another Black teenager” [*P9*]. Culturally-specific representations therefore offered a meaningful way to increase a sense of belonging for adolescents’ depression experiences, thereby informing their perceptions about who should seek help for mental illness.

#### Creating an accessible blueprint

Across this desire for relatability, participants broadly wanted a clear blueprint on the practicalities of seeking help and reassurance. Participants recommended including more experiential details, specific steps for seeking help, or resources available for adolescents, casting the video interventions as a vehicle not just for legitimacy but also for explicit instructions on next steps. The intervention's format was accessible: Participants responded positively to the study's one-minute video, noting that it was an appropriate length for their attention spans, but still sufficiently empowering. Many found the concept of sharing one's struggle as admirable and believed that hearing how peers received help is encouraging for others experiencing depression: “It also gave me the courage to ask for help whenever I need it because, you know, a young person telling the world that they need help and they asked for it… It's very encouraging for other teens too” [*P19*]. Consequently, with adjustments for authenticity and instructiveness, participants viewed videos as an accessible, motivating intervention.

## Discussion

Our qualitative study aimed to gather input from adolescents to develop a brief video-based intervention aimed at reducing depression stigma and increasing help-seeking. Twenty adolescents aged 15–18 participated in semi-structured interviews that included watching a one-minute sample video, commenting on stigma, identity, systemic barriers, and school's role as significant factors influencing their attitudes toward mental health care. Schools figured as pivotal sources of mental health resources and education, with adolescents wanting more comprehensive support systems and peer-based programs. Additionally, participants highlighted the influence of social media videos, emphasizing the need for authentic, culturally-tailored narratives that provide a clear, relatable blueprint for help-seeking. Adolescents suggested these videos could validate their experiences and guide them towards appropriate mental health resources, with a call for more detailed content that resonates with their own struggles and demographics. To our knowledge, this is the first study to seek input from teenagers to inform the development of a brief social-contact-based video intervention to reduce stigma and increase help-seeking.

The themes of barriers to help-seeking—including questions of identity, social, parental and peer stigma, and systemic barriers—corroborate extant literature (5, 19). Villatoro et al. reported that adolescents were more likely to make help-seeking recommendations for peers with mental health problems than they were to seek help for a problem of their own (5). This aligns with our findings that adolescents desire better depression acceptance and resources yet still struggle with self-stigma. Villatoro et al. additionally found that adolescents who self-labeled as having mental health struggles were more likely to engage in help-seeking behavior (5), aligning with our finding that validation and internal acceptance of a depression identity is soothing to participants and increases willingness to seek help. A systematic review by Aguirre et al. described that stigma, family beliefs, perceived need for self-sufficiency, and structural barriers like staffing and availability are key factors that prevent adolescents from seeking help (19), echoing our participants’ remarks. Simultaneously, despite this substantial research on adolescents’ perceived barriers to help-seeking, there remains a gap in literature regarding specific guidance for intervention to overcome these obstacles. Our study contributes to addressing this gap.

Participants offered rich commentary on the role of schools, viewing schools as critical yet underutilized resources for mental health support. They perceived school systems as capable of providing both mental health education and concrete support resources and noted that these responsibilities are well-within schools’ purview. Among the few studies that have explored adolescents’ perceptions of the role of school, Wilson and Deane (25) demonstrate that building trust and relationships with “gatekeeper adults” (i.e., parents and teachers) was a key factor for help-seeking in focus groups of high school students. Similarly, our study reveals adolescents’ desire for clear, actionable guidance from adults and peers alike on how to seek mental health assistance. This includes a need for detailed experiential insights, concrete steps for seeking help, and readily accessible resources. These same needs can be targeted not just at school, meeting demand for in-person supports, but also virtually, through interventions like social media videos.

Regarding school contributions, the fact that participants mentioned incorporating parents into possible school mental health literacy initiatives underscored the significance of parental stigma vs. acceptance as an area for future consideration. Interestingly, participants did not link video interventions specifically to school settings nor to parental attitudes, raising opportunities for future research exploring potential synergy between these areas of adolescent life, given the prominence of family, school, and the Internet in many adolescents’ lives.

Our findings identify social media videos as a potentially effective medium for encouraging help-seeking behaviors, underscoring the value of relatable and accessible peer support. A systematic review by Goodwin and Behan (26) found limited evidence to whether and how social media videos could influence help-seeking behavior and highlighted the need for more rigorous research. Another study by Schnyder et al (27), showed the association between stigma and help-seeking and urged the development of interventions to reduce negative personal attitudes specifically by strengthening beliefs in the treatability of mental illness. Our study answers this call by illustrating adolescent perceptions of taking action for mental health, be it through peers, parents, or school, as well as offering insight into how to craft social media videos in an authentic way. Participants were acutely sensitive to the language and demeanor of the actress in this study's video stimulus, and our qualitative data provides many examples of adolescent-specific syntax, including the frequent appearance of “like,” which has been described as a “youth-solidarity indicator” (28). As done in prior studies of identity-specific video interventions for depression stigma (16), future social media videos should take care to craft narratives with similar linguistic solidarity.

Simultaneously, the findings from our study prompt a deeper consideration of the role of social media in adolescent mental health, suggesting possible implications for future interventions and policies. Social media serves as a double-edged sword. On one hand, it offers a range of benefits for children and adolescents, providing a platform for community building, self-expression, and access to information, particularly for marginalized groups such as racial, ethnic, and sexual and gender minorities (29–31). In addition, research suggests that social media-based and other digitally based mental health interventions may help some young people by promoting help-seeking behaviors and serving as a gateway to initiating mental health care (32–35). On the other hand, the potential risks associated with social media cannot be overlooked. Our participants’ concerns about confidentiality, peer judgment, and perceived inauthenticity echo concerns raised in recent literature, including the Surgeon General’s Advisory about the harmful effects of social media on young people's mental health (36). Excessive or inappropriate use of these platforms has been linked to increased depression and anxiety, as noted in studies by Alonzo et al. and others (37). This dichotomy underscores the urgent need for targeted research and development of interventions that can create safer and healthier online environments for young people. Because our study identified that adolescents seek similar remedies from proposed interventions at school and through social media videos (namely, connection to a relatable peer community, better mental health education, and directions on how to access care), there is opportunity for potential synergy between school initiatives and social media, which could additionally provide a protective measure against over- or mis-use of social media due to the incorporation of adult guidance through school.

In light of these findings, our study could serve as a steppingstone towards addressing the Surgeon General’s call to action. It lays the groundwork for the creation of brief, social contact-based video interventions focused on destigmatizing depression and promoting openness to treatment. By incorporating the insights gained from our adolescent participants, these interventions can be designed to be culturally sensitive, age-appropriate, and engaging, thus maximizing their effectiveness. Furthermore, this approach aligns with the broader public health objective to foster safe and supportive online spaces that reinforce positive mental health outcomes for youth. As such, our study proposes practical, actionable steps that can be taken to mitigate the adverse effects of social media on adolescent mental health while harnessing its potential benefits.

### Strengths, limitations and future directions

Our study raises several considerations for future research based on strengths and limitations. First, the themes articulated in each interview align with our previous studies and other existing literature, suggesting good external validity. Moreover, though our sample spanned only 20 participants, we reached theoretical sufficiency by the end of all interviews, indicating sufficient depth of qualitative analysis (22). Our sample also differs from the general population in its demographic makeup. According to analysis of US Census data, the racial identities of adolescents are reported as 14% Black, 50% white, 26% Hispanic, 5% Asian, and 5% multiracial (38), compared to 45% Black, 40% white, and 15% multiracial in our sample (these three participants cumulatively reported white, Black, Middle Eastern, Hispanic, and Asian identities). The representation in our sample is an overall strength that increases representation of racially minoritized perspectives in the literature, although future research would benefit from further representation of Hispanic and Asian identities, as our sample does exhibit a gap in representation for these demographics.

Relatedly, although our results identified salient interactions between identity and mental health, one limitation is that our interview guide did not explicitly assess for the impact of identity, and further elaboration could have deepened our assessment of adolescent gender, race, and other cultural identities on depression stigma and help-seeking behavior. Future mixed-methods studies could expand upon our preliminary qualitative results to more deeply explore the nuances of identity on help-seeking behavior via brief video interventions, with particular attention to underrepresented demographic groups as above. Simultaneously, given that the study was advertised as an opportunity to discuss depression and stigma, this may have created a self-selection bias in our participant sample.

Third, the reliance on self-reporting may be biased by efforts to please the interviewers, although both the interview guide and interviewer styles were flexible and open in efforts to minimize desirability bias. Fourth, youth participation in this study was contingent upon parental consent: Given that this study explores a stigmatized topic and stigma itself, requiring parental consent may pose a barrier to participation for those who do not feel safe discussing stigmatized topics with their parents or others. This consideration is not unique to our study, but future work may nonetheless target ways to explore experiences of stigma and help-seeking in populations of minors without parental stigma as a barrier.

## Conclusion

In conclusion, the findings highlight the importance of addressing systemic barriers, enhancing school-based mental health support, and utilizing social media platforms for culturally relevant and engaging mental health content. These insights have significant implications for developing targeted interventions that can effectively reduce stigma and encourage help-seeking among adolescents with depression. Specifically, well-designed video interventions can serve as a “blueprint” for adolescents to acknowledge, accept, and de-stigmatize their experiences of depression and to follow peers’ steps in seeking help. These findings are particularly timely and relevant in light of the Surgeon General's recent advisory on youth mental health, which calls for urgent action in these areas. Our study provides a roadmap and input that can assist in the development of brief video interventions, which have the potential to improve mental health outcomes for young people.

## Data Availability

The raw data supporting the conclusions of this article will be made available by the authors, without undue reservation.

## Appendix 1—Interview Guide

Thank you for speaking with me today. This interview will consist of questions about your thoughts on mental illness, specifically depression, and accessing care. Do you have any questions before we start?

Part 1: Experiences of Stigma and barriers to care.

1. What kind of beliefs do you think people in society have towards people with mental illness (and specifically depression)?
2. How have experiences of prejudice/discrimination created obstacles to your own mental health care?
3. What does your family or culture think of depression? Does the way your family or culture think of depression affect the way you think of depression?
4. Many young people who suffer from depression avoid treatment and seeking help. Why do think teenagers with depression avoid that?
5. What kind of interventions would be helpful with changing attitudes and accessing care (and specifically depression)?

Part 2: Perspectives on a Video Intervention.

1. How do you feel when you hear other people's personal stories of mental illness and recovery and specifically depression)?
  a. Please tell us about examples where you have heard other people's personal stories (e.g., group therapy, support groups, online community, watching videos)
  b. What aspects of these stories do you find helpful?
  c. What aspects of these stories do you find unhelpful?
  d. Thinking back to specific experiences, did anything change for you after hearing someone else's story?
2. Do you remember any change in your perception/behavior following a video you watched? (or any other content)
3. We are now going to watch a short video that we created to reduce public discrimination toward people with depression. We are curious to hear what you think about it.
  a. What do you think about the video presenter?
  b. How do you feel about the video presenter?
  c. What did you think about the length of the video—too long or too short?
4. We want to create a similar video that is targeted toward people with depression.
  a. What should we consider including in this video?
  b. What would you find encouraging?

Part 3: School.

1. How does high-school impact your mental health, if at all?
2. Tell us a little about any resources that exist at your school for mental health support. If there aren't any, what resources do you think could be helpful?
3. What responsibility do you think schools have to support students’ mental health, if at all?

Part 4: Wrap Up.

1. If you could tell other people with depression one thing that you think would reduce stigma and promote their empowerment, what would it be?
2. Is there anything we didn't discuss today that you would like to share?
3. As we mentioned, we are collecting some minimal demographic data. Would you be comfortable sharing your race, gender, and age?
